# The effects of long-term exposure to microgravity and body orientation relative to gravity on perceived traveled distance

**DOI:** 10.1038/s41526-024-00376-6

**Published:** 2024-03-13

**Authors:** Björn Jörges, Nils Bury, Meaghan McManus, Ambika Bansal, Robert S. Allison, Michael Jenkin, Laurence R. Harris

**Affiliations:** 1 Center for Vision Research, York University, 4700 Keele Street, Toronto, ON M3J 1P3 Canada; 2 Institute of Visual Computing, Hochschule Bonn-Rhein-Sieg, Grantham-Allee 20, St. Augustin, 53757 Germany; 3 Department of Experimental Psychology, Justus Liebig University Giessen, Otto-Behaghel-Strasse 10F, 35394 Giessen, Germany

**Keywords:** Psychology, Neuroscience

## Abstract

Self-motion perception is a multi-sensory process that involves visual, vestibular, and other cues. When perception of self-motion is induced using only visual motion, vestibular cues indicate that the body remains stationary, which may bias an observer’s perception. When lowering the precision of the vestibular cue by for example, lying down or by adapting to microgravity, these biases may decrease, accompanied by a decrease in precision. To test this hypothesis, we used a move-to-target task in virtual reality. Astronauts and Earth-based controls were shown a target at a range of simulated distances. After the target disappeared, forward self-motion was induced by optic flow. Participants indicated when they thought they had arrived at the target’s previously seen location. Astronauts completed the task on Earth (supine and sitting upright) prior to space travel, early and late in space, and early and late after landing. Controls completed the experiment on Earth using a similar regime with a supine posture used to simulate being in space. While variability was similar across all conditions, the supine posture led to significantly higher gains (target distance/perceived travel distance) than the sitting posture for the astronauts pre-flight and early post-flight but not late post-flight. No difference was detected between the astronauts’ performance on Earth and onboard the ISS, indicating that judgments of traveled distance were largely unaffected by long-term exposure to microgravity. Overall, this constitutes mixed evidence as to whether non-visual cues to travel distance are integrated with relevant visual cues when self-motion is simulated using optic flow alone.

## Introduction

When we walk down a street, our motion relative to objects in the environment, such as trees, lamp posts, and buildings, generates a pattern of visual motion known as optic flow^1,2^. Optic flow over the whole field provides an important source of information that helps us keep track of our motion through the environment. Optic flow alone can provide information as to how far^3–6^, how fast^7,8^, and in which direction^9,10^ we have traveled. However, optic flow is not usually the only cue to self-motion: the vestibular system monitors linear accelerations of the head, which can be double integrated to provide a noisy estimate of traveled distance^11,12^. Somatosensory cues and efference copy also contribute during active self-motion^13,14^, and auditory^15,16^, and haptic cues^17^ have been shown to contribute as well. In many scenarios, the cues from different sensory modalities are integrated according to their relative precision^18^.

During natural self-motion, these cues are in agreement, but during visually induced self-motion of a stationary observer (vection), vestibular, somatosensory, and proprioceptive cues indicate no motion. Might these other cues moderate or restrain the self-motion percept? The vestibular system is always “on”, indicating accelerations due to head movement as well as the acceleration of gravity. Here, we manipulate the vestibular system’s background steady state by comparing performance while upright and supine on Earth and by testing astronauts in the microgravity of the International Space Station (ISS).

When upright, the direction of gravity aligns with acceleration associated with up and down head movement, and when supine, with forward and backward (sagittal) head movement. A supine posture is associated with an overestimation of perceived travel distance when participants feel they are upright^19^. The latency of vection onset is reduced when supine compared to upright^20–22^, and the magnitude of vection is larger^22^. Therefore, we compared the perception of self-motion when supine with that when upright and looked for any differences before and after spaceflight.

Locomotion on the International Space Station (ISS) is very different from moving around on Earth. The effective lack of gravity means that astronauts typically glide from one module to another, and their otoliths are usually “unloaded” and stimulated only by their own acceleration^23^. Oman et al.^24^ speculated that in microgravity environments, people might increase the weight given to visual cues, which may alter their experience of vection. They reported that the vection onset time of astronauts on Neurolab was reduced and that astronauts subjectively felt significantly faster motion while in microgravity compared to their pre-flight baseline. These observations were supported by Allison et al.^25^, who also found a decrease in vection onset latency when viewing smooth and jittering visual motion during brief periods of microgravity created by parabolic flight compared to when tested on Earth. Adding jitter makes the optic flow more like what would be experienced during normal walking as opposed to the smooth gliding movement experienced by astronauts moving around within the ISS. Overall, these rare studies suggest that while free-floating in microgravity, people may be more sensitive to visual information for perceived self-motion due to an increased weighting of visual cues (c.f., Harris et al.^26^). While the microgravity-related disruptions in the vestibular cue onboard the ISS are accompanied by somatosensory changes, a recent study by Bury et al.^27^ found no significant difference in perceived traveled distance between a neutrally buoyant condition underwater and the control condition on Earth. Any changes in self-motion perception to microgravity exposure could therefore be attributed to the vestibular cue.

It is an open question how exactly visual, vestibular and other cues are integrated to develop the perception of self-motion – particularly when self-motion is evoked purely by optic flow^28^. Often, during multisensory integration, cues are weighted according to their relative reliabilities^18,29^. Thus, if visual cues indicating forward self-motion are equally reliable as vestibular cues indicating no self-motion, then the final percept should be, on average, midway between the two. Conversely, if one estimate is less precise, the final percept would be biased more towards the other cue. This is relevant here because the precision of vestibular cues depends on posture, with vestibular information being less precise when participants are lying down, either on their side or supine^30–36^. If the perception of self-motion visual and vestibular cues are indeed integrated according to their relative reliabilities, a decrease in the reliability of the vestibular cue or disruption of normal vestibular signaling, such as in microgravity, might then lead to an increase in perceived self-motion, as measured—for example—through perceived traveled distance. McManus and Harris^19^ indeed found this expected increase in perceived traveled distance for supine observers (compared to when they were upright), particularly when they experienced a visual reorientation illusion in which they misperceived that they were upright.

**Fig. 1:**
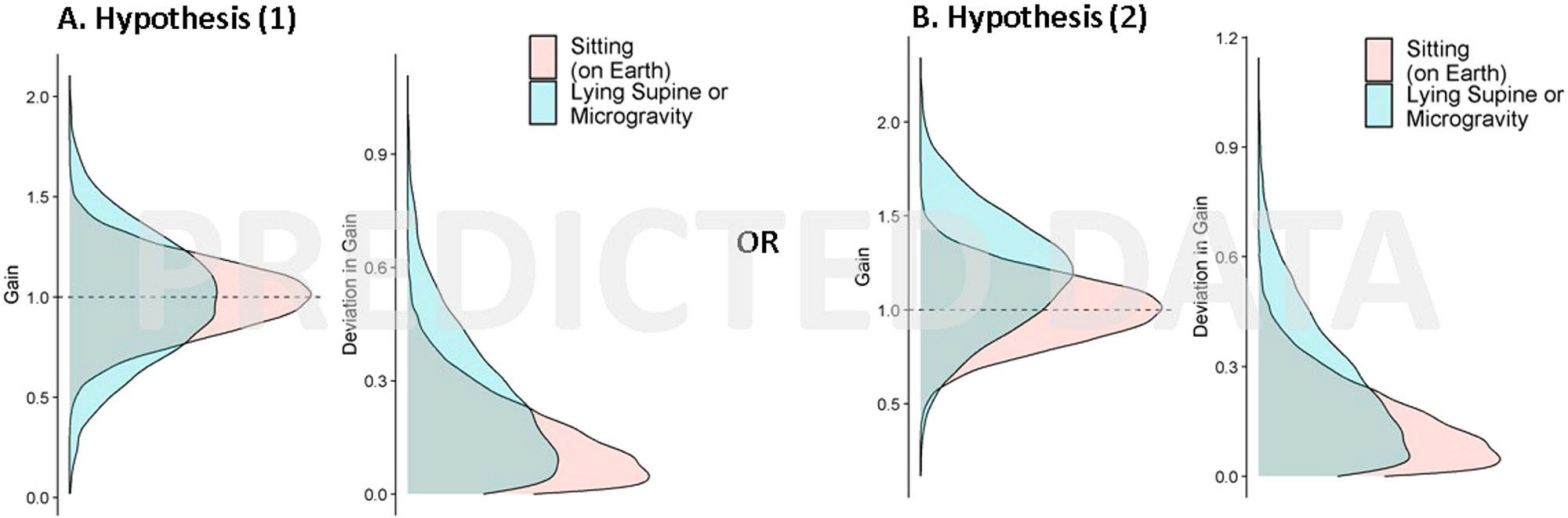
Predictions. Predicted distributions of the self-motion gains (as a measure of accuracy, see methods for definitions) and the self-motion deviations (as a measure of precision, higher deviations mean lower precision) for the two postures sitting upright (red) and supine (blue). Exposure to microgravity was hypothesized to show the same trends as when supine. Either the gain may become noisier (Hypothesis 1, see panel A) or both noisier and with a higher gain (Hypothesis 2, see panel B). Different panels depict the expected data when Hypotheses 1a and 1b are true (**A**) or when Hypotheses 2a and 2b are true (**B**).

We hypothesized that (Hypothesis 1a) the alteration of Earth-normal vestibular cues when in microgravity and (Hypothesis 1b) a decrease in reliability in the vestibular cue when supine in Earth-normal conditions would both result in higher variability in the judgments of travel distance (see Fig. 1, left side). We further hypothesized that if visual cues about forward self-motion are integrated with vestibular and somatosensory cues signaling that the body is at rest, (Hypothesis 2a) the alteration of Earth-normal vestibular cues in microgravity and (Hypothesis 2b) less reliable vestibular cues when supine should bias the global self-motion percept less and therefore when in microgravity or when supine, participants would require less visual motion to perceive they had traveled a given distance compared to when sitting upright (higher gains, Fig. 1, right side).

## Results

### Astronauts

**Fig. 2:**
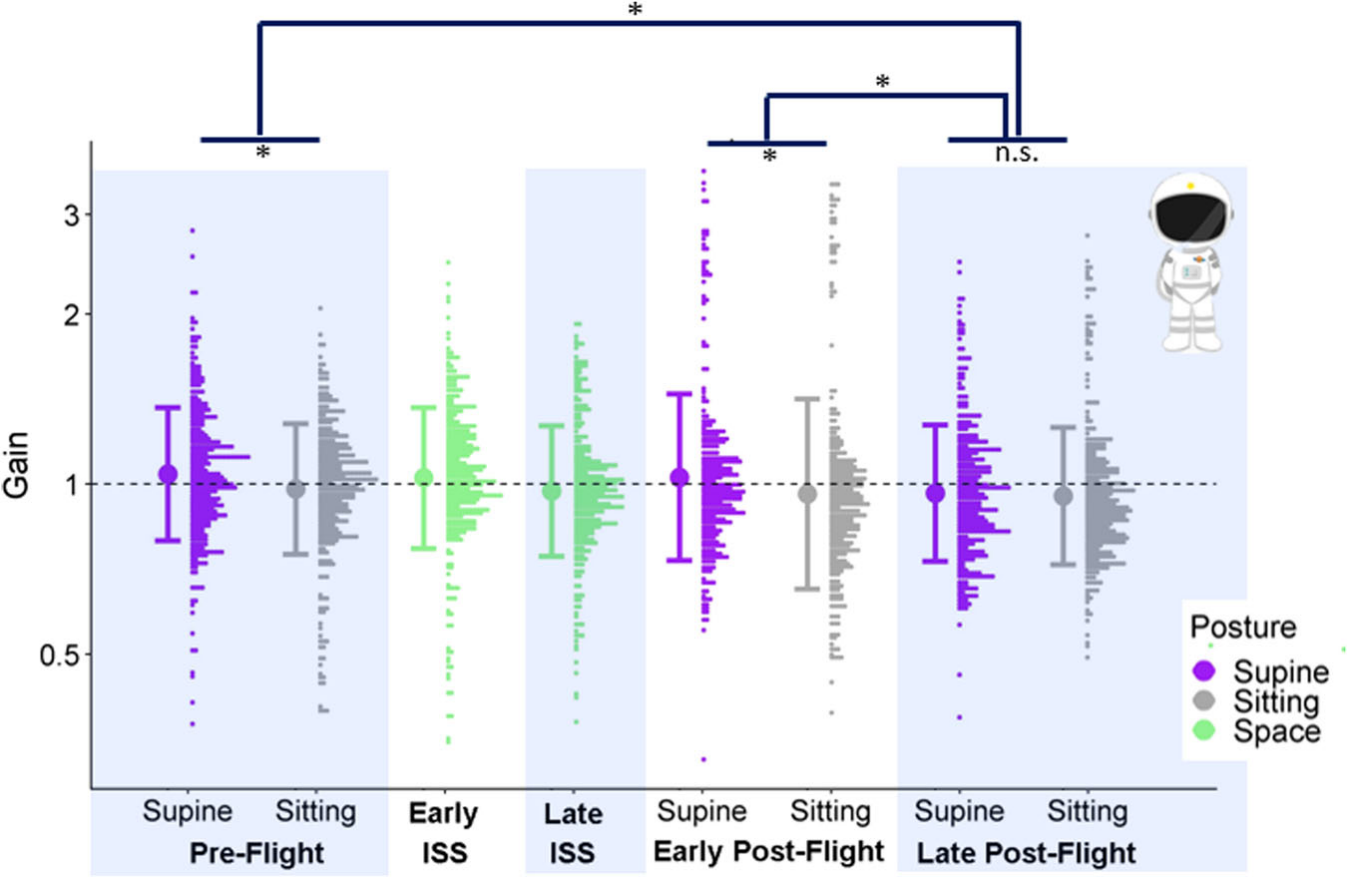
Accuracy data for astronauts. Full distributions of the astronauts’ gains for the different test sessions and postures were generated at a bin width of 0.0175 and plotted on a log scale. The postures are color-coded (purple for supine, gray for sitting, and green for in-space sessions). The bold dot to the left of each distribution indicates the mean across all participants for the corresponding test session and posture, and the bars correspond to ±1 standard deviation. Asterisks indicate significant differences in the means at a significance level of 0.05.

For the astronauts, we found that the supine posture led to significantly higher gains than the sitting posture in the Pre-Flight (by 0.07, 95% CI = [0.02, 0.1]) and Early Post-Flight sessions (by 0.05, 95% CI = [0.01, 0.09]), but not in the Late Post-Flight sessions. In line with this finding, we found significant interactions between Session and Posture, indicating that the difference between Sitting and Supine was smaller in the Late Post-Flight session than in both the Pre-Flight session (by −0.05, 95% CI = [−0.09, −0.02]) and the Early Post-Flight session (by 0.04, 95% CI = [−0.08, −0.001]). No other significant differences were found. See Fig. 2.

**Fig. 3:**
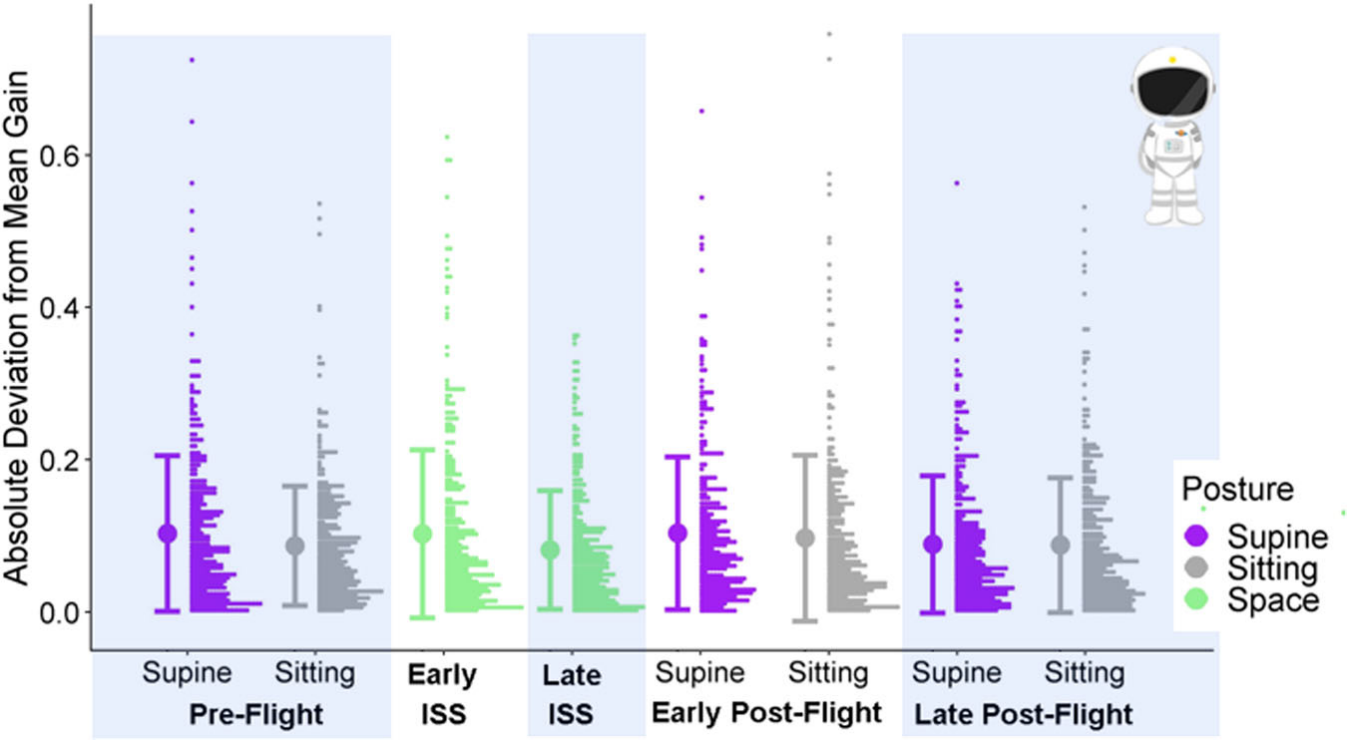
Precision data for astronauts. Full distributions of the astronauts’ absolute deviations from the mean gain for each test session and posture were generated at a bin width of 0.075. The postures are color-coded (purple for supine, gray for sitting, and green for in-space sessions). The bold dot to the left of each distribution indicates the mean across all participants for the corresponding test session and posture, and the bars correspond to ±1 standard deviation.

In the precision model for the astronauts, we found that larger mean ratios were associated with higher deviations from the mean (with a coefficient of 0.12, 95% CI = [0.07, 0.17]). However, we found no significant differences for any of the contrasts we assessed (see Fig. 3).

### Controls

**Fig. 4:**
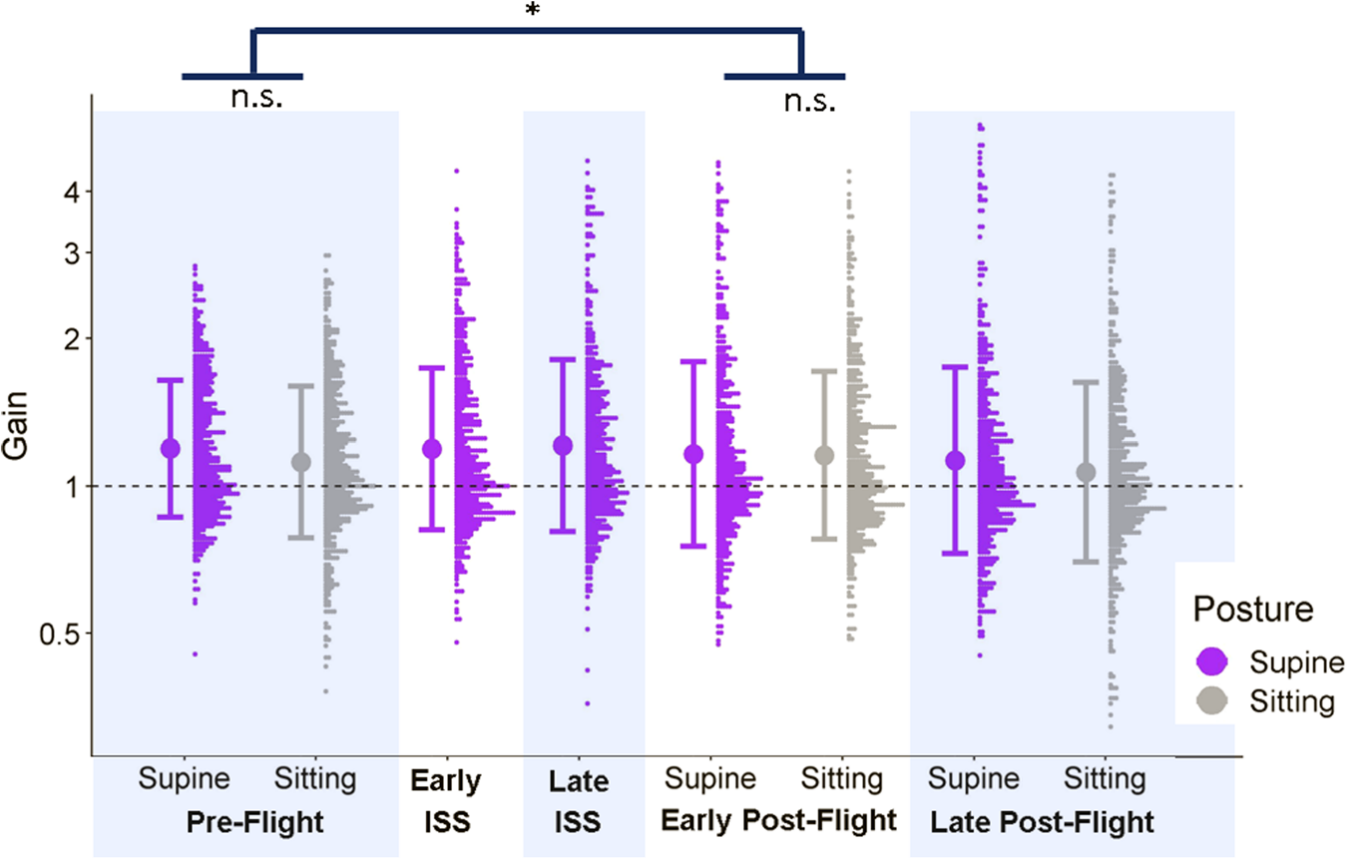
Accuracy data for controls. Full distributions of the controls’ gains for the different test sessions and postures, generated with a bin width of 0.0175 gains and plotted on a log scale. The postures are color-coded (purple for supine and gray for sitting). For the control participants, the “ISS” sessions were completed on Earth in the supine position. The bold dot to the left of each distribution indicates the mean across all participants for the corresponding test session and posture, and the bars correspond to ±1 standard deviation.

We did not find significant differences in gains between the test sessions or the postures within any of the test sessions. However, we did find a significant interaction between Posture in the Pre-Flight and Early Post-Flight sessions: gains for Sitting relative to Supine were significantly higher in the Early Post-Flight session than in the Pre-Flight session (by 0.06, 95% CI = [0.03, 0.1]). See Fig. 4.

**Fig. 5:**
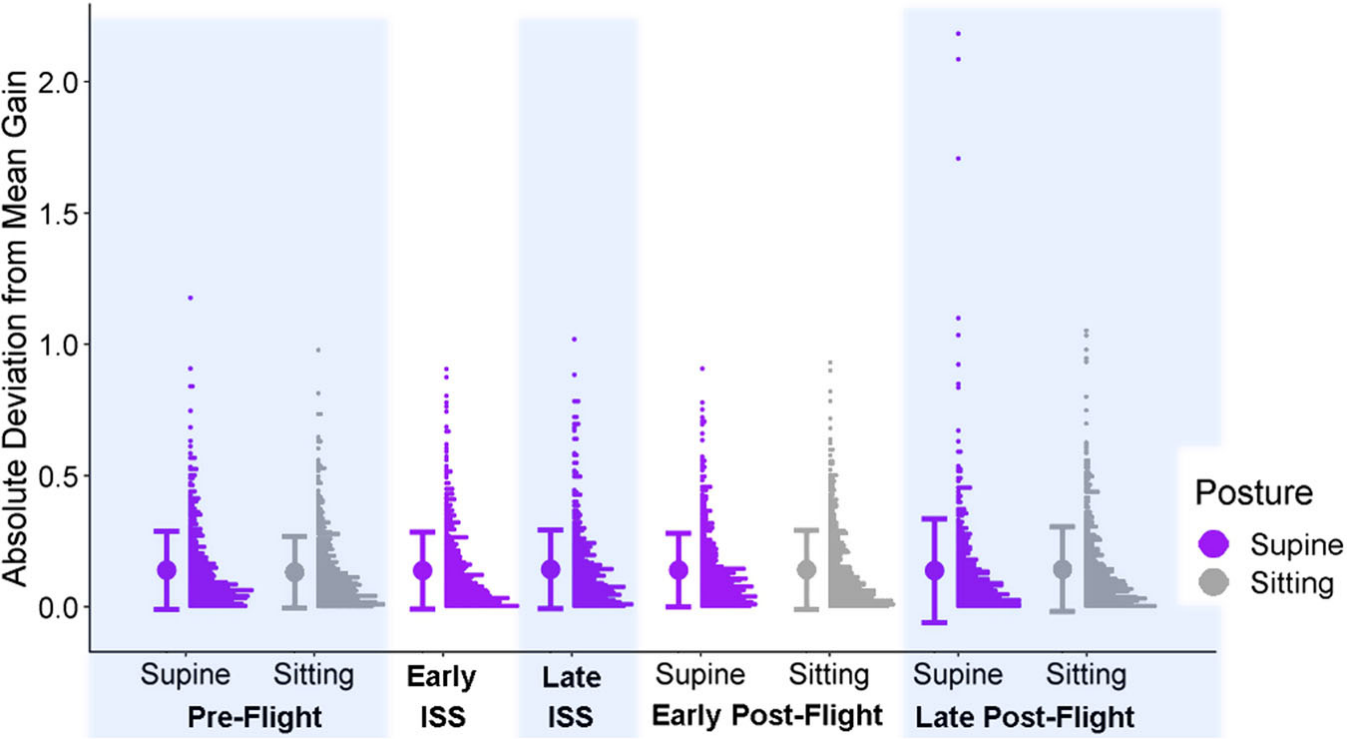
Precision data for controls. Full distributions of the controls’ absolute deviations from the mean gain for the different test sessions and postures were generated at a bin width of 0.075. The postures are color-coded (purple for supine and gray for sitting). For the control participants, the “ISS” sessions were completed on Earth in the supine position. The bold dot to the left of each distribution indicates the mean across all participants for the corresponding test session and posture, and the bars correspond to ±1 standard deviation.

In the controls’ precision model, larger mean ratios were associated with higher deviations from the mean (with a slope of 0.13, 95% CI = [0.1, 0.15]). None of the other contrasts (between any of the sessions, postures, or their interactions) were significantly different from zero. See Fig. 5.

We also explored potential sex and/or gender differences in our participants’ reactions to both microgravity and postural manipulation. However, these analyses were largely inconclusive (see Supplemental Material).

## Discussion

**Fig. 6:**
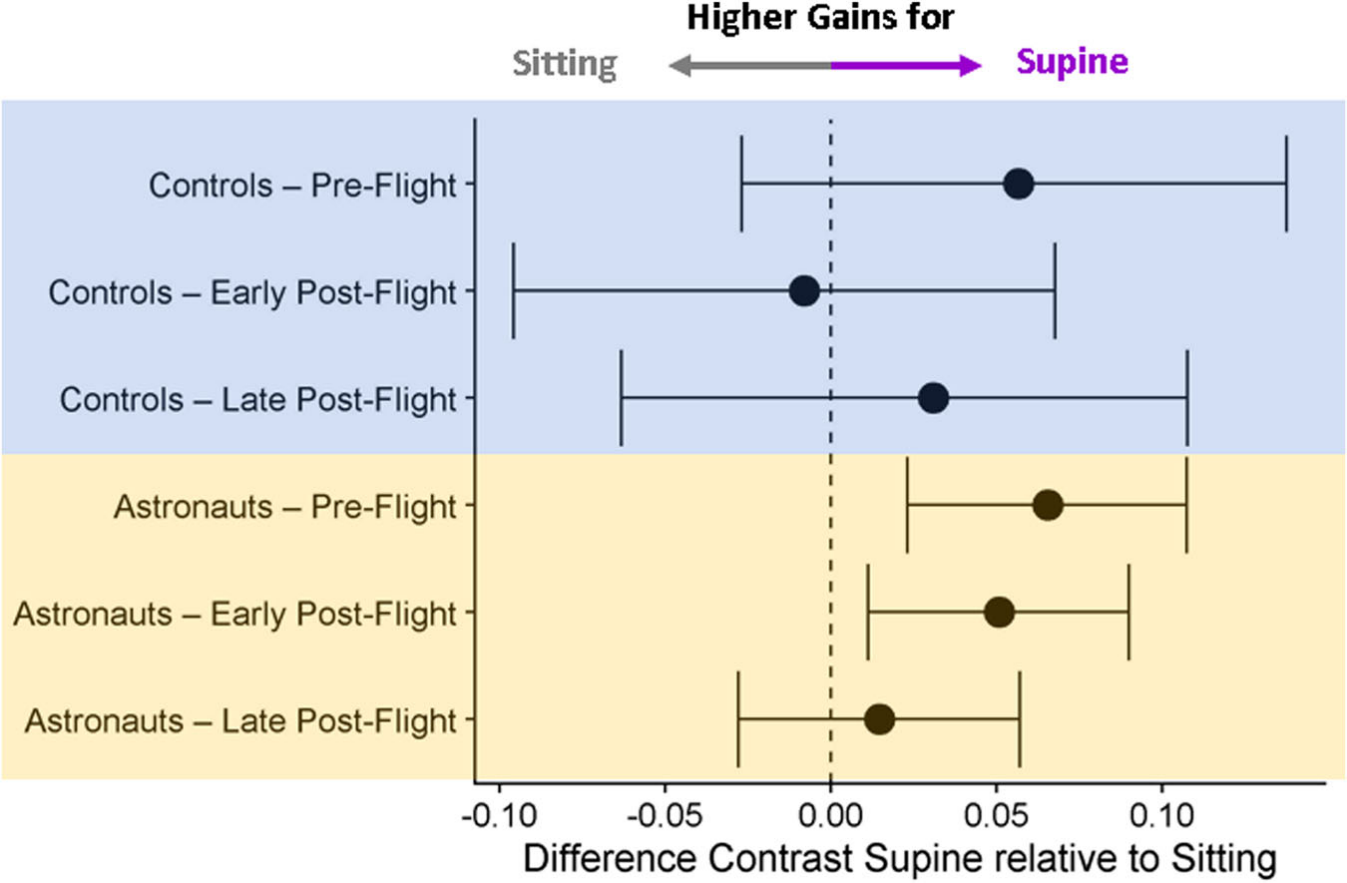
Difference contrasts for Supine vs Sitting. Fitted difference contrast parameters from the linear mixed model analysis (dots) for Supine vs Sitting for all test sessions in which participants performed the task in both postures, along with bootstrapped 95% confidence intervals. The dashed vertical line indicates no difference between Supine and Sitting.

Overall, on Earth, we found that our astronaut cohort needed less optic flow when supine than when sitting upright to feel they had traveled the same distance in Pre-Flight and Early Post-Flight but not in Late Post-Flight. No such differences were found in the controls, while neither group showed any significant change in variability in response to the postural manipulation (Fig. 6).

Astronauts’ performance of their estimate of the distance of self-motion, either in terms of accuracy or precision, did not change significantly in response to microgravity exposure.

We tested two hypotheses as to how posture might affect performance in the perception of traveled distance: Hypothesis 1: performance in the move-to-target task would be more variable when supine or in microgravity than in the sitting upright condition on Earth, and Hypothesis 2: participants would show an enhancement in their use of visual cues both when supine and in microgravity, which would show as undershooting in the move-to-target task relative to when sitting upright on Earth.

We found no significant differences in precision between sitting and upright but did find a small but robust overestimation of perceived traveled distance when supine compared to when sitting upright in Pre-Flight and Early Post-Flight (but not in Late Post-Flight) in the astronauts. (see Fig. 2). The controls’ performance was not affected significantly by the manipulation. Our data thus supported Hypothesis 2 to some extent while not supporting Hypothesis 1. We have no suggestions for the mechanism by which such long-term changes in the effect of posture might arise, but this does seem to be a minor consequence of microgravity exposure and is reminiscent of long-term changes in perceived orientation^26^ and in the perception of size^37^ following missions on the International Space Station.

Hypothesis 1 was predicated on findings that vestibular cues are noisier when lying supine than when upright and disrupted in microgravity^31–36^, therefore, maximum likelihood estimates of the combined percept should be less precise as the contribution of the vestibular sense degrades. This was not found (see Fig. 3). This may be explained in two ways: First, it was possible to perform our task based on visual information alone, for which reason vestibular cues might just have been discarded. However, we did find a bias towards larger gains in the supine posture in some sessions (Fig. 2), which suggests that non-visual cues can be integrated into the final percept. A second, more likely explanation is that other sources of error, such as variability in the processing of optic flow or the perception of distance, might dominate the total variability in our task, eclipsing any contribution of vestibular noise. Another caveat is that we did not test any astronaut earlier than three days after their arrival onboard the International Space Station. It is possible that the vestibular system had already adapted to its new environment within that period.

In support of our Hypothesis 2, we found that some participants needed to travel a shorter distance to reach where they thought the target was when supine than when sitting upright for the same target distance, that is, they had higher gains. This indicates that visual and non-visual cues were integrated despite the discrepancy between them. Both when sitting and when supine, vestibular and somatosensory cues signaled that the body was at rest, whereas the visual cue signaled forward self-motion at an acceleration of 0.8 m/s^2^. Vestibular precision is decreased when supine in comparison to upright^34^. If vestibular cues signaling that the body is at rest become less precise when supine, they should also bias the global self-motion estimate less than when sitting upright. A lower precision when supine may therefore have led to differences in accuracy between the postures. Two caveats are in order with regard to this explanation: First, as stated in the previous paragraph, we did not find evidence for a difference in precision between the two postures. Second, there is ample evidence that when two multisensory cues diverge as strongly as in our case, one is usually disregarded in favor of the other, a process referred to as “robust integration”^38^ or “segregation”^29^, as opposed to “fusion” in which two similar-enough cues are integrated. Overall, the hypothesis that postural changes in perceived self-motion as a result of posture changes are caused by differences in vestibular sensitivity thus requires further examination.

As an alternative to our Hypothesis 2, a misinterpretation of the otolith stimulation in the supine posture as acceleration rather than tilt might underlie the pattern we have observed to some extent in our study. Otolith signals when supine are similar to those expected when the body is upright and accelerating forward. While somatosensory and visual cues usually disambiguate these vestibular cues, this disambiguation process might be incomplete leading to the interpretation that the vestibular cue is indicating forward acceleration. This might bias the observer to overestimate self-motion, leading them to require less optic flow to have felt they had traveled the same distance (corresponding to the higher visual gains we observed while supine). While the dataset used for this paper does not allow us to adjudicate between these hypotheses, a recent study from our lab tested participants in standing upright, supine, and prone conditions. The “tilt as acceleration” hypothesis would predict that participants should need less optic flow to perceive they had traveled the same distance in the supine condition than in the upright condition, while they should require more optic flow in the prone condition. However, McManus and Harris^19^ found that participants required less optic flow both when supine and when prone, which is incompatible with the “tilt as acceleration” hypothesis.

A supine posture can lead to biased distance perception^39,40^. Thus posture-dependent biases in the perceived distance of our targets could be a confound in this study. If such a bias carried over to the perception of traveled distance in the present task, participants would need less optic flow when supine than when in an upright posture. While this is what we found, it is unlikely on a conceptual level that biases in distance perception induce biases in the perception of traveled distance: If, for example, due to the well-documented underestimation of distance in virtual reality settings, participants underestimate their initial distance to the target, they should also scale down the distance to other objects in the environment that induce optic flow (such as the walls in our experiment). Any biases in distance perception should thus cancel out.

This study tackled the question of whether body posture influences human perception of self-motion and distance. We found some evidence that the same amount of optic flow can elicit the sensation of having traveled further when supine versus when sitting upright, that is, optic flow is more effective at eliciting a sense of self-motion when supine. This constitutes evidence that visual and non-visual cues are at least partially integrated even when self-motion is presented only visually. However, we did not find any significant differences between performance on Earth and in the microgravity of the ISS, suggesting that vestibular cues play a minor role, if any, in the estimation of visually presented self-motion.

On a more applied level, this shows that astronauts are unlikely to be exposed to dangers due to an unusual perception of traveled distance when in space, for example, when sensitive equipment and machinery must be operated manually and in a visually guided fashion in the absence of gravity. While we found inconclusive evidence as to whether men and women performed differently overall or reacted differently to our manipulations (see supplemental material), this makes it unlikely that any sex/gender differences would be significant.

## Methods

### Participants

We obtained written informed consent from all participants. This investigation was approved by the local ethics committee at York University as well as by the Canadian Space Agency (CSA), NASA, JAXA, and the ESA. All participants had normal or corrected-to-normal vision and reported no balance issues. During experiments, all participants wore their habitual contact lenses or eyeglasses.

We tested a cohort of 15 astronauts (8 women, 7 men). Three of these participants completed only the first test session (Pre-Flight), either because their space flight was delayed until after the intended sample size (complete data sets from 6 women and 6 men) had already been reached (1 woman, 1 man) or because their second test session could not be completed within 6 days of launch (1 woman). The incomplete datasets were excluded from the analysis. The 12 participants (6 men, 6 women) that finished all test sessions had a mean age of 42.6 years (SD = 5.4 years, 38.7 years for women and 46.6 years for men).

We recruited 22 participants to form the control group (11 women, 11 men). Two participants dropped out after the first or during the second test session due to excessive motion sickness such that only 20 participants (10 men and 10 women) finished all experimental sessions. There were no other reports of motion- or cyber-sickness. Data from the dropouts were excluded from the analysis. The control group’s mean age at recruitment was 42.6 years (SD = 7.2 years, 43.9 years for women and 41.3 years for men).

### Apparatus

We used an Oculus Rift CV1 virtual headset with a diagonal visual field of about 110° to present the stimuli. It has a resolution of 1080 × 1200 pixels per eye and a refresh rate of 90 Hz. Stimuli were programmed in Unity. Head-tracking was disabled, and stimuli were presented without disparity cues. We used an HP IDS DSC 4D Z15 Base NB PC with an Intel Core i7-4810MQ Quad Core and an NVIDIA Quadro K610M graphics card to control the experiment and generate the visual environment. All responses were given with a 3 G Green Globe Co Ltd. (FDM-G62 P) finger mouse.

### Stimuli and procedure

**Fig. 7:**
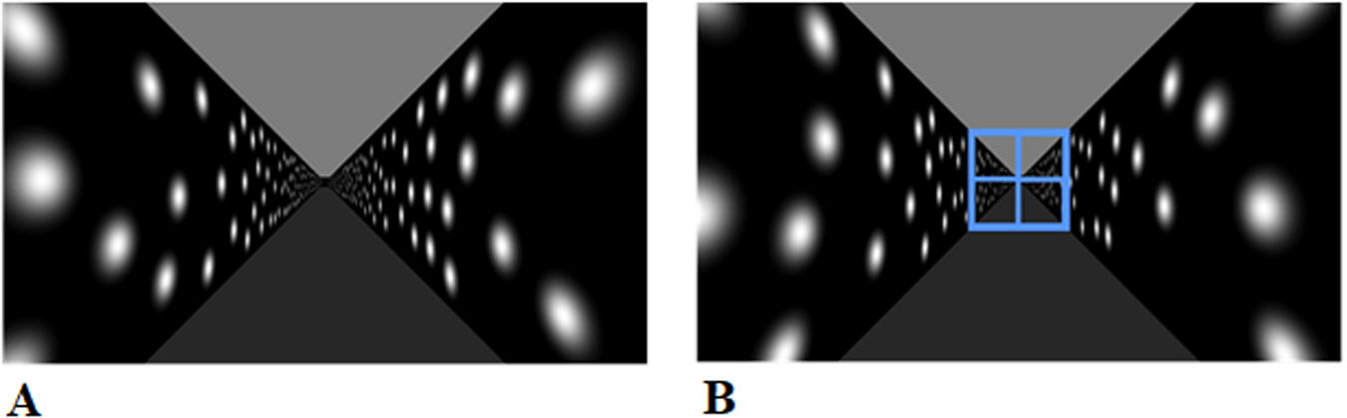
Screenshots from the experiment. **A** Screenshot from the hallway in which the participants were immersed. **B** The target is shown at the beginning of each trial.

We immersed the participants in a virtual reality hallway environment (Fig. 7A) that extended ahead of them. The hallway was simulated as 3.3 m tall and 3.3 m wide, and the participants’ viewpoint was fixed in the center at a viewing height of 1.65 m. Light spots rendered at random locations on the walls provided the optic flow information. The light spots were Gaussian blobs and were rendered to ±2 sigma. The blobs disappeared and reappeared at random intervals and locations such that they could not easily be used as fixed landmarks.

Participants viewed a target that filled the whole hallway and which was presented at various simulated distances ahead of them (6–24 m in steps of 2 m, Fig. 7B). Participants were asked to view the target and build an estimate of the egocentric distance to the target. Once the participant had built an appropriate estimate of the distance to the target, they started the trial by pressing a button on the finger mouse. The target was extinguished, and the participant was subjected to optic flow, indicating self-motion toward the previously presented target. Participants were asked to indicate when they had reached the position of the target viewed previously by pressing a button on the finger mouse. For each trial, the visually simulated distance traveled (the amount of optic flow presented) was recorded, as was the point at which the button was pressed. Between trials, the display was reset for the start of the next trial. Participants were not provided with any feedback about their performance (i.e., whether they stopped at the correct location of the target or not). Each trial used the same simulated acceleration (0.8 m/s^2^), and each target distance was presented three times. A short sequence from the experiment can be viewed on Open Science Foundation: https://osf.io/k7yt8.

### Test sessions

The astronauts were tested on five occasions: once before launch (Pre-Flight), within the first 3–6 days after launch (Early ISS), about 87 days after launch (Late ISS), within the first 3–6 days after return (Early Post-Flight), and finally about 85 days after return (Late Post-Flight) (see Tables in Supplemental material). While on Earth, we tested the astronauts in two postures: sitting upright and lying supine. We counterbalanced the posture with which participants started each session. In space (onboard the International Space Station), the astronauts were floating freely, but a backrest attached to the cabin prevented them from drifting while conducting the experiment. Here, data was only collected in this one orientation.

Approximately matching the timing of the astronaut’s data collection sessions, the control participants were tested at similar intervals to the astronauts over a period of roughly a year (see Table 2 in Supplemental Material). Data collection for the controls occurred in 2019 and 2020 before the COVID-19 pandemic. For the second and third test sessions (the Early ISS and Late Post-Flight analogs), participants performed the experiment lying supine only, as the closer on-Earth analog for space.

### Data analysis

Data analysis was performed using R 4.2.2. All data and the R code used for analysis can be found on Open Science Foundation (https://osf.io/pvmyh/).

For data analysis, we first computed the visual gain for each trial. The visual gain is computed as:

1 $${\rm{Gain}}=\frac{\rm{Target}\,{distance}}{\rm{Perceived}\,{traveled}\,{distance}}$$

A gain value above one corresponds to participants stopping too early or undershooting for a specific target distance. Stopping early (a higher gain) would suggest that the optic flow was more effective in making the participant believe they had traveled the given distance. A gain value below one corresponds to participants stopping too late or overshooting, that is, the same amount of optic flow led them to believe they had traveled less far. This, in turn, would mean they had to travel further to perceive they had reached the target.

We then proceeded to perform an outlier analysis by excluding all trials where the participant did not press a button on the corresponding trial. For the controls, one female participant had a mean gain of more than three standard deviations above the mean across participants and was excluded for this reason. For the remaining participants, we further removed all data points more than three standard deviations above or below the mean for each session and the target distance of their cohort (astronauts or controls). This led to the exclusion of 58 out of the total of 4400 data points (1.3%) for the controls and to the exclusion of 30 out of the total of 3030 trials (1%) for the astronauts.

To determine precision in responses, we computed the deviation from the mean for each trial for each condition (session, target distance, and posture) and participant separately. For precision analysis only, we excluded those conditions where (due to the initial outlier analysis) only one value was left, making it impossible to calculate the deviation from the mean. By this criterion, three additional data points were excluded for the controls (0.06%) and one for the astronauts (0.03%).

For statistical analysis, we used linear mixed modeling as implemented in the lme4 package^41^ for R^42^. To determine the appropriate model structures, we started with a maximal model^43^ that included random slopes for all relevant independent variables (Posture—a categorical variable with the values “Sitting”, “Supine”, and “Space”, Test Session—a categorical variable with the values “Pre-Flight”, “Early ISS”, “Late ISS”, “Early Post-Flight” and “Late Post-Flight”, and Target Distance—as a categorical variable with the values 6 m, 8 m, …, 24 m) for the grouping variable Participant. Since we were interested in the effects of microgravity and posture on accuracy and precision, we used Posture, Test Session, and their interaction as fixed effects, and since having a variable represented only as a random effect but not as a fixed effect can make parameter estimates unreliable, we also set Target Distance as a fixed effect. The dependent variables were either the gains (for accuracy) or the deviations (for precision). Models were fitted separately for controls and astronauts.

For accuracy, we fitted the following models in the Wilkinson & Rogers^44^ formalism for the astronauts and the controls.

2 $$\begin{array}{l}{\rm{Gain}} \sim {\rm{Test}}\; {\rm{Session}}* {\rm{Posture}}+{\rm{Target}}\; {\rm{Distance}}+ ({\rm{Posture}}+{\rm{Session}}+{\rm{Target}}\; {\rm{Distance|Participant}})\end{array}$$

We then computed bootstrapped confidence intervals at an alpha level of 0.05 as implemented in the confint function from base R to assess statistical significance.

To assess differences in precision, we employed the analyses detailed for accuracy, but with the deviations as the dependent variable. Since, due to Weber’s Law, higher gains are expected to lead to proportionally higher variability, we also added the mean gains per condition as additional fixed effects, thus testing for whether Posture and Session explained variability beyond what was expected by accuracy differences. The model was thus specified as follows:

3 $$\begin{array}{l}{\rm{Deviation}} \sim {\rm{Mean}}+{\rm{Session}}* {\rm{Posture}}+{\rm{Target}}\; {\rm{Distance}}+({\rm{Posture}}+{\rm{Session}}+{\rm{Target}}\; {\rm{Distance|Participant}})\end{array}$$

### Reporting summary

Further information on research design is available in the Nature Research Reporting Summary linked to this article.

### Supplementary information

Supplemental material

Reporting Summary

## Data Availability

All data, the code used for analysis, as well as a video of the stimulus can be found on Open Science Foundation (https://osf.io/pvmyh/).
