# Supplementary material for: The effects of long-term exposure to microgravity and body orientation relative to gravity on perceived traveled distance
Source: NPJ Microgravity. 2024 Mar 13;10:28. doi: 10.1038/s41526-024-00376-6 (PMC10937641; doi:10.1038/s41526-024-00376-6)
Supplement: Supplementary file 1 — Supplemental material [file 41526_2024_376_MOESM1_ESM.pdf]

The Effects of Long-Term Exposure to Microgravity and Body Orientation Relative to Gravity on Perceived Traveled Distance

SUPPLEMENTAL MATERIAL

Björn Jörges[1]*, Nils Bury[1 2], Meaghan McManus[1 3], Ambika Bansal[1], Robert S. Allison[1], Michael Jenkin[1], Laurence R. Harris*[1]

[1]Center for Vision Research, York University, 4700 Keele Street, Toronto, ON M3J 1P3, Canada

[2]Institute of Visual Computing, Hochschule Bonn-Rhein-Sieg, Grantham-Allee 20, 53757 St. Augustin, Germany

[3]Department of Experimental Psychology, Justus Liebig University Giessen, Otto-Behaghel-Strasse 10F, 35394 Giessen, Germany

*Corresponding Author

Keywords:

Self-motion, microgravity, astronauts, visuo-vestibular integration, virtual reality, posture.

As space flight programs start to move past some of their biases in astronaut recruitment – like the fact that NASA only selected young, white, male astronauts during its first 20 years – it becomes increasingly important that microgravity studies ensure representative participant samples. As a small step in this direction, one of the explicit goals of the VECTION project, funded by the Canadian Space Agency, was to assess whether exposure to microgravity affected male and female astronauts differently.

Sex and/or gender differences have been reported for both visual and vestibular percepts. In an exploratory study, Shaqiri and colleagues[1] found differences between women and men in six out of fifteen visual tasks, however there did not appear to be any specific pattern or grouping between the tasks in which such differences were found. A study by Barnett-Cowan et al.[2] found that, compared to men, women rely more on vision and are more variable in their determination of the subjective visual vertical when they are upright. When lying right side down there was no differences between the men and women. In terms of vestibular function, Herpers and colleagues[3] reported that five out of five men in their sample experienced the somatogravic illusion, while only one out of five women did. There also seems to be a lower prevalence of vestibular disorders in men than in women [4]. Hormonal differences are given as tentative root cause for differences in vestibular dysfunction, although the authors note that these disparities persist even for post-menopausal women and age-matched men. This casts some doubts on a purely hormonal explanation. Sociological differences between women and men that may be responsible for these disparities were not discussed.

When it comes to sex/gender differences in the perception of self-motion and vection specifically, there are mixed findings. Some studies report that women rate vection as more convincing than men[5] and women have shorter onset latencies for circular vection[6]. However, other studies have failed to find differences in latency[5] , and few studies have looked at sex and/or gender differences for the perception of linear self-motion.

While there certainly are biological differences ("sex differences") between women and men, these have often been overstated in psychological research[7]. Sociological differences between these two gender groups ("gender differences") may be more important to observed differences in performance. For instance, any gender differences in the use of video games or use virtual reality headsets might lead to participants being more comfortable performing experiments using joysticks, hand-held controllers, or virtual reality headsets. There are also socio-anatomical factors such as the fact that many common head-mounted displays do not accommodate interpupillary distances low enough for a large number of women[8]. Lastly, it is important to note that research on sex and gender differences has almost exclusively focused on cis women and cis men, neglecting gender diversity in terms of gender modality (cis or trans) and in terms of other genders beyond women and men.

Since for the gender effects, we were also interested in potentially confirming the absence of an effect, we fitted Bayesian Linear Mixed Models using the brms package [9] for R that were equivalent to the main models used to assess our main hypotheses (Equations 1 and 2), except that we added the Sex/Gender of the participant as well as its interactions with Posture and Session as well as the triple interaction between Sex/Gender, Posture and Session as fixed effects. For the accuracy model, we used a Gaussian distribution, while we used a shifted log normal distribution for the precision model due to the heavily right-skewed nature of the deviations.

Gain ~ Test Session * Posture * Gender + Target Distance + (Posture + Session + Target Distance | Participant) (S1)

Deviation ~ Gain + Test Session * Posture * Gender + Target Distance + (Posture + Session + Target Distance | Participant) (S2)

We then used the hypothesis function to compute the Bayes Factors. We set up the analysis such that Bayes Factors below 1 signify evidence for the null hypothesis (i.e., evidence for the absence of a difference), while Bayes Factors above 1 signify evidence for the alternative hypothesis (i.e., evidence for the presence of a difference). When interpreting the Bayes Factors, we follow Andraszewicz' and their colleagues' guidelines[10], where 1 corresponds to "no evidence", 1 to 3 to anecdotal evidence for the alternative hypothesis, 3 to 10 to moderate evidence for the alternative hypothesis, 0.33 to 1 to anecdotal evidence for the null hypothesis and 0.1 to 0.33 to moderate evidence for the null hypothesis.

For the main effect of sex/gender on accuracy (gains), we found a Bayes Factor of 0.41 ("anecdotal evidence" as per Andraszewicz[10]) in favor of the hypothesis that there is no difference between men and women. Further, when looking at the interaction between sex/gender and posture, we obtained a Bayes Factor of 0.15 (moderate evidence) in favor of the hypothesis that there were no differences in how men and women reacted to the postural manipulation. We further found Bayes Factors of 0.8 and 0.8 for the interaction between sex/gender and the sessions Early ISS and Late Post-Flight, indicating inconclusive evidence as to whether men and women reacted differently to microgravity exposure. Gains are plotted separately for men and women in Supplementary Figure 1.

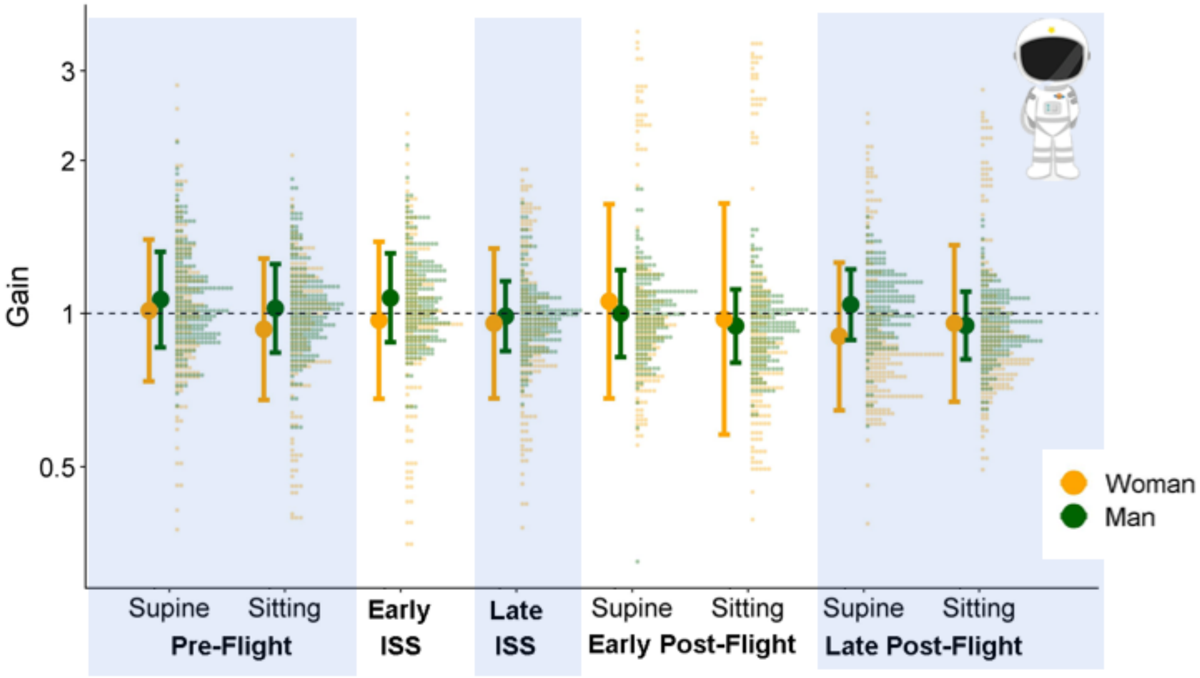

Supplementary Figure 1:

Accuracy data for male/female astronauts

Full distributions of the astronauts' gains for the different test sessions and postures, generated at a bin width of 0.0175 and plotted on a log scale. The participants' sex/gender is color coded (men green, women orange). The bold dots to the left of each distribution indicates the mean values for the men (green) and women (orange) for the corresponding test session and posture. The bars correspond to +/-1 standard deviation.

We found a Bayes Factor of 0.45 for the main effect of sex/gender, indicating inconclusive evidence for the absence of a difference between men and women, and a Bayes Factor of 0.16 (moderate evidence) for the hypothesis that men and women reacted equally to the postural manipulation. Similar to the case for accuracy, we found inconclusive evidence as to whether men and women reacted differently to microgravity exposure with Bayes Factors of 0.8 and 0.8 respectively. Deviations in gains are plotted separately for men and women in Supplementary Figure 2.

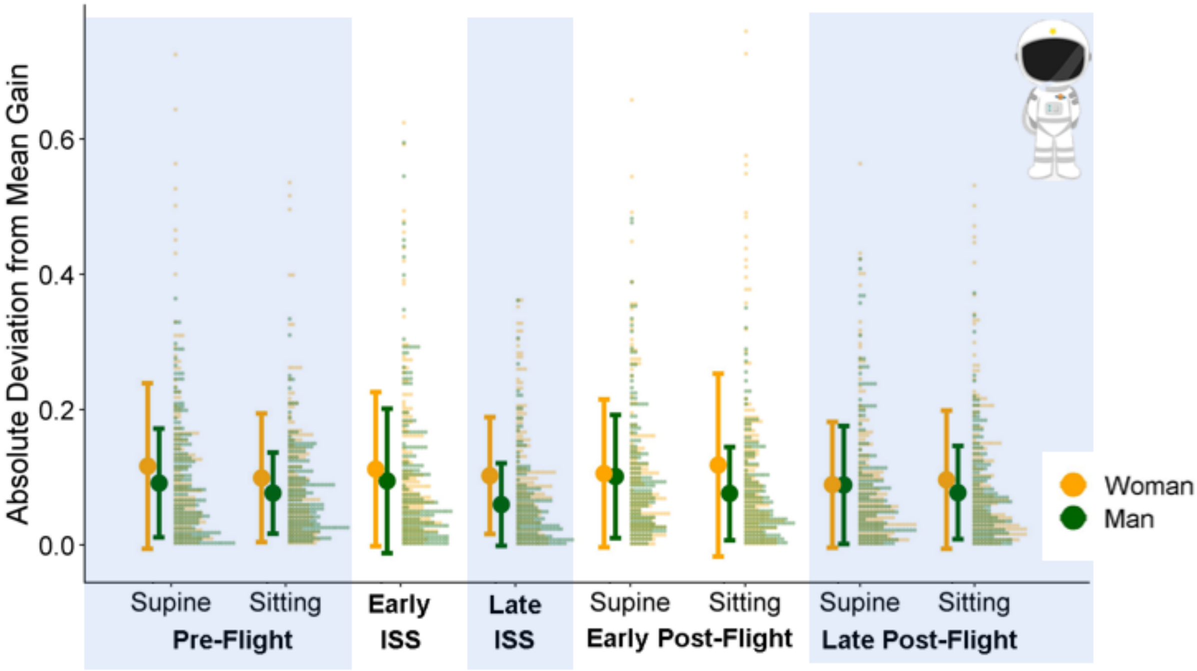

Supplementary Figure 2:

Precision data for male/female astronauts

Full distributions of the astronauts' deviations from the mean gain for the different test sessions and postures, generated at a bin width of 0.075. The participants' sex/gender is color coded (men green, women orange). The bold dots to the left of each distribution indicates the mean values for the men (green) and women (orange) for the corresponding test session and posture. The bars correspond to +/-1 standard deviation.

In the controls, we found inconclusive evidence (Bayes factor of 0.92) as to whether the gains of men and women differed across all test sessions. Further, we found moderate evidence (Bayes factor of 0.23) that women and men reacted in the same way to the postural manipulation. Gains are plotted separately for men and women in Supplementary Figure 3.

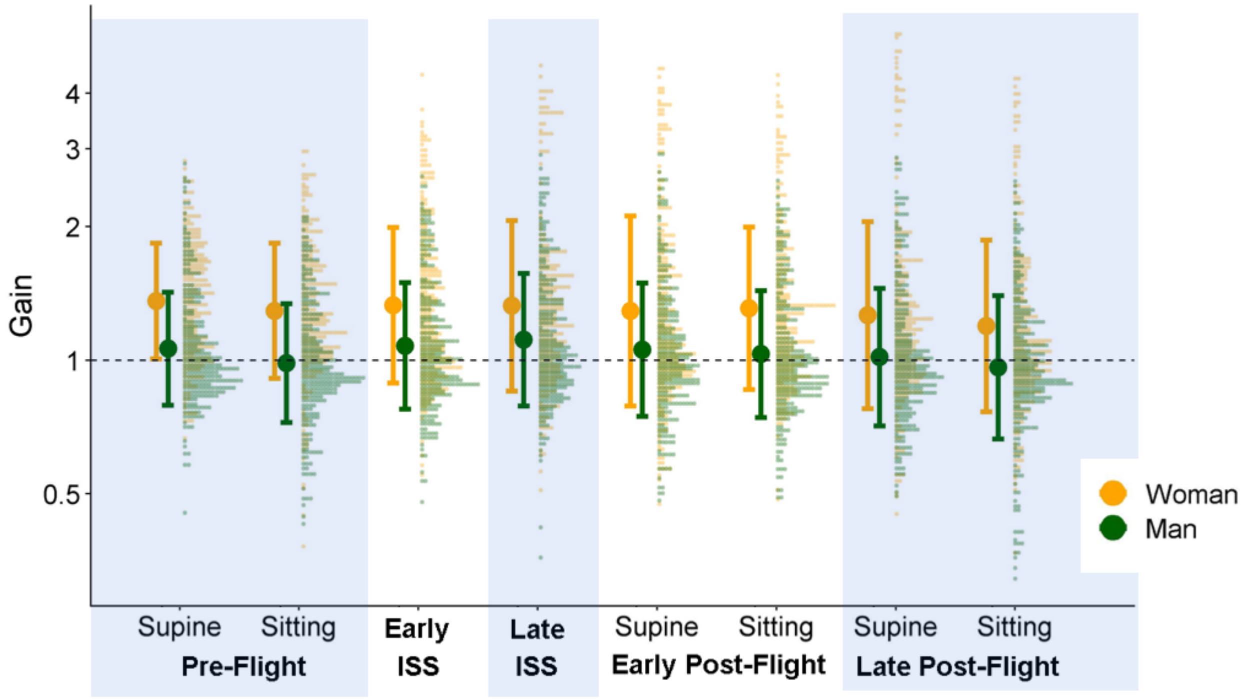

Supplementary Figure 3:

Accuracy data for male/female controls

Full distributions of the controls' gains for the different test sessions and postures, generated at a bin width of 0.0175 and plotted on a log scale. The participants' sex/gender is color coded (men green, women orange). For the control participants the "ISS" sessions were completed on Earth in the supine position. The bold dots to the left of each distribution indicates the mean values for the men (green) and women (orange) for the corresponding test session and posture. The bars correspond to +/-1 standard deviation.

Precision – We found inconclusive evidence that the deviations in gain differed between female and male controls (Bayes factor of 1.06). We further found anecdotal evidence (Bayes factor of 0.39) that women and men reacted equally to the postural manipulation. Deviations in gain are plotted separately for men and women in Supplementary Figure 4.

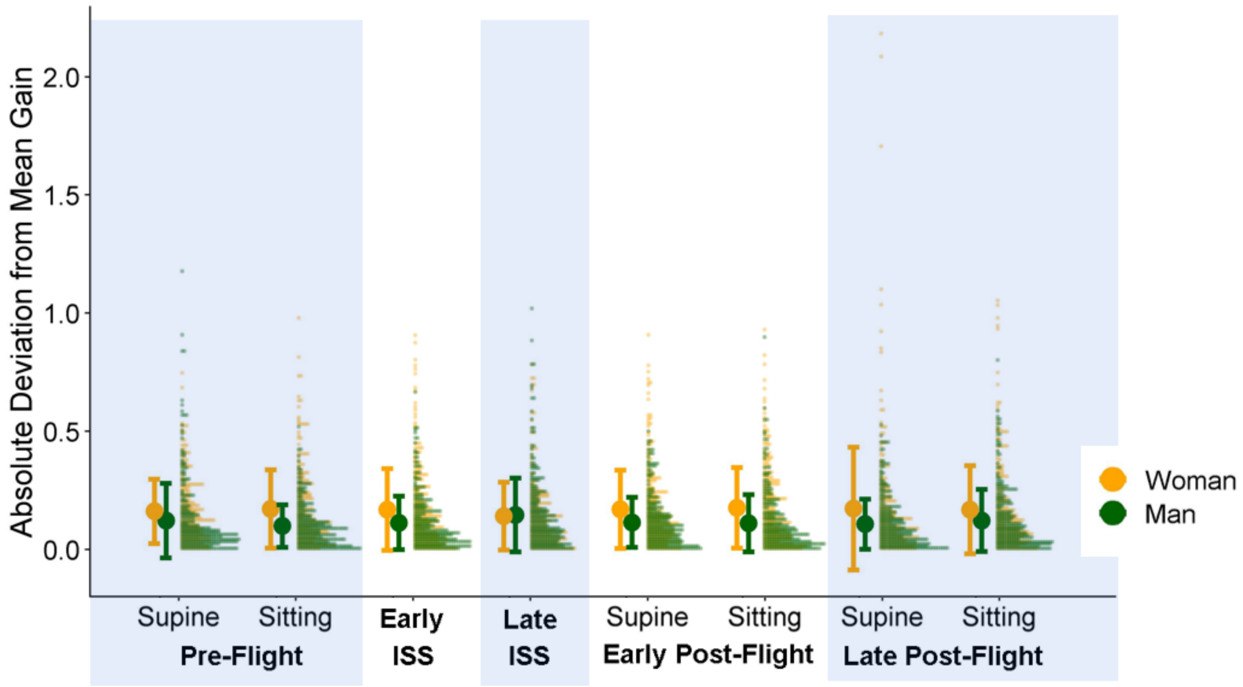

Supplementary Figure 4:

Precision data for male/female controls

Full distributions of the controls' deviations from the mean gain for the different test sessions and postures, generated at a bin width of 0.075. The participants' sex/gender is color coded (men green, women orange). For the control participants the "ISS" sessions were completed on Earth in the supine position. The bold dots to the left of each distribution indicates the mean values for the men (green) and women (orange) for the corresponding test session and posture. The bars correspond to +/-1 standard deviation.

Overall, we find either inconclusive evidence regarding sex/gender differences in the perception of self-motion or moderate evidence for the absence of such differences. More specifically, we found moderate evidence in both cohorts (astronauts and controls) that men and women reacted equally to the postural manipulation in terms of accuracy. Evidence was largely inconclusive when it came to overall main effect differences between men and women, as well as regarding whether women and men reacted differently to microgravity exposure.

SUPPLEMENTAL REFERENCES

1. Shaqiri, A. *et al.* Sex-related differences in vision are heterogeneous. *Sci. Rep.* **8**, 1–10 (2018).
2. Barnett-Cowan, M., Dyde, R. T., Thompson, C. & Harris, L. R. Multisensory determinants of orientation perception: task-specific sex differences. *Eur. J. Neurosci.* **31**, 1899–907 (2010).
3. Herpers, R. *et al.* The somatogravic illusion during centrifugation: sex differences. *Front. Physiol.* **9**, 24–27 (2018).
4. Smith, P. F., Agrawa, Y. & Darlington, C. L. Sexual dimorphism in vestibular function and dysfunction. *J. Neurophysiol.* **121**, 2379–2391 (2019).
5. Riecke, B. E., Schulte-Pelkum, J., Avraamides, M. N., Heyde, M. V. D. & Bülthoff, H. H. Cognitive factors can influence self-motion perception (vection) in virtual reality. *ACM Trans. Appl. Percept.* **3**, 194–216 (2006).
6. Darlington, C. L. & Smith, P. F. Further evidence for gender differences in circularvection. *J Vestib Res-Equilib Orientat* **8**, 151–153 (1998).
7. Hyde, J. S., Bigler, R. S., Joel, D., Tate, C. C. & van Anders, S. M. The future of sex and gender in psychology: Five challenges to the gender binary. *Am. Psychol.* **74**, 171–193 (2019).
8. Stanney, K., Fidopiastis, C. & Foster, L. Virtual Reality Is Sexist: But It Does Not Have to Be. *Front. Robot. AI* **7**, (2020).

9. Bürkner, P. C. Advanced Bayesian multilevel modeling with the R package brms. *R J.* **10**, 395–411 (2018).

10. Andraszewicz, S. *et al.* An Introduction to Bayesian Hypothesis Testing for Management Research. *J. Manag.* **41**, 521–543 (2015).

SUPPLEMENTAL TABLES

#	Gender	PRE-FLIGHT	Early ISS	Late ISS	Early POST-FLIGHT	Late POST-FLIGHT
1	M	L-87	L+3	L+86	R+6	R+62
2	F	L-87	L+3	L+86	R+6	R+62
3	F	L-183	L+5	L+95	R+6	R+180
4	M	L -186	L+5	L+87	R+6	R+124
5	M	L-184	L+5	L+87	R+6	R+54
6	M	L-204	L+4	L+92	R+6	R+62
7	F	L-111	L+6	L+92	R+4	R+54
8	M	L-264	L+3	L+85	R+5	R+46
9	M	L-165	L+3	L+83	R+3	R+57
10	F	L-147	L+3	L+84	R+4	R+165
11	F	L-247	L+3	L+83	R+6	R+61
12	F	L-141	L+3	L+83	R+4	R+87
	#M: 6<br>#F: 6	Ø: L-167d<br>±56.5d	Ø: L+3.8d<br>±1.1d	Ø:<br>L+86.9d<br>±4d	Ø: R+5.1d<br>±1.1d	Ø:<br>R+84.5d<br>±46d

Supplementary Table 1: The timing of each test session (on Earth and on-orbit test sessions) for each astronaut relative to launch (L) and return (R), along with aggregate statistics (mean and standard deviation) of the time between the test session and launch.

#	Gender	PRE-FLIGHT	Early ISS (simulated)	Late ISS (simulated)	Early POST-FLIGHT	Late POST-FLIGHT
1	f	L-142	L+3	L+93	R+3	R+59
2	f	L-126	L+3	L+98	R+3	R+58
3	m	L-41	L+3	L+91	R+3	R+56
4	m	L-28	L+3	L+86	R+3	R+59
5	m	L-114	L+3	L+90	R+3	R+57
6	f	L-82	L+3	L+93	R+3	R+65
7	m	L-118	L+3	L+91	R+3	R+57
8	f	L-118	L+3	L+91	R+3	R+59
9	f	L-122	L+3	L+88	R+3	R+55
10	m	L-114	L+3	L+96	R+3	R+59
11	f	L-81	L+3	L+93	R+3	R+62
12	f	L-54	L+3	L+81	R+3	R+52
13	m	L-122	L+3	L+99	R+3	R+66
14	f	L-23	L+3	L+87	R+3	R+64
15	f	L-101	L+3	L+88	R+3	R+59
16	f	L-46	L+3	L+92	R+3	R+53
17	m	-	-	-	-	-
18	m	L-91	L+3	L+91	R+3	R+60
19	m	L-20	L+3	L+94	R+3	R+59
20	m	L-42	L+3	L+88	R+3	R+58
21	m	L-44	L+3	L+96	R+3	R+66
22	f	-	-	-	-	-
	#m: 10<br>#f: 10	Ø: L-81.45d ±40.3d	Ø: L+3	Ø: L+91.3d ±4.3d	Ø: R+3	Ø: R+59.2d ±3.9d

Supplementary Table 2: This table gives an overview of our schedule for the participants of our control group. The bottom line shows the average of days relative to their simulated launch into space (L) which is considered as 'day 0' with the standard deviation below, or relative to the simulated return from space (R).
