# Supplementary material for: The effects of long-term exposure to microgravity and body orientation relative to gravity on perceived traveled distance
Source: NPJ Microgravity. 2024 Mar 13;10:28. doi: 10.1038/s41526-024-00376-6 (PMC10937641; doi:10.1038/s41526-024-00376-6)
Supplement: Supplementary file 2 — Reporting Summary [file 41526_2024_376_MOESM2_ESM.pdf]

nature portfolio

Corresponding author(s): Björn Jörges

Last updated by author(s): Feb 11, 2024

Reporting Summary

Nature Portfolio wishes to improve the reproducibility of the work that we publish. This form provides structure for consistency and transparency in reporting. For further information on Nature Portfolio policies, see our Editorial Policies and the Editorial Policy Checklist.

Statistics

For all statistical analyses, confirm that the following items are present in the figure legend, table legend, main text, or Methods section.

n/a	Confirmed	
☐	☒	The exact sample size (*n*) for each experimental group/condition, given as a discrete number and unit of measurement
☐	☒	A statement on whether measurements were taken from distinct samples or whether the same sample was measured repeatedly
☐	☒	The statistical test(s) used AND whether they are one- or two-sided<br>*Only common tests should be described solely by name; describe more complex techniques in the Methods section.*
☒	☐	A description of all covariates tested
☒	☐	A description of any assumptions or corrections, such as tests of normality and adjustment for multiple comparisons
☐	☒	A full description of the statistical parameters including central tendency (e.g. means) or other basic estimates (e.g. regression coefficient) AND variation (e.g. standard deviation) or associated estimates of uncertainty (e.g. confidence intervals)
☐	☒	For null hypothesis testing, the test statistic (e.g. *F*, *t*, *r*) with confidence intervals, effect sizes, degrees of freedom and *P* value noted<br>*Give P values as exact values whenever suitable.*
☐	☒	For Bayesian analysis, information on the choice of priors and Markov chain Monte Carlo settings
☒	☐	For hierarchical and complex designs, identification of the appropriate level for tests and full reporting of outcomes
☒	☐	Estimates of effect sizes (e.g. Cohen's *d*, Pearson's *r*), indicating how they were calculated

Our web collection on statistics for biologists contains articles on many of the points above.

Software and code

Policy information about availability of computer code

Data collection	Stimuli were collected using a custom program built in Unity.
Data analysis	Data analysis was performed using R (version 4.2.2)

For manuscripts utilizing custom algorithms or software that are central to the research but not yet described in published literature, software must be made available to editors and reviewers. We strongly encourage code deposition in a community repository (e.g. GitHub). See the Nature Portfolio guidelines for submitting code & software for further information.

Data

Policy information about availability of data

All manuscripts must include a data availability statement. This statement should provide the following information, where applicable:

- Accession codes, unique identifiers, or web links for publicly available datasets
- A description of any restrictions on data availability
- For clinical datasets or third party data, please ensure that the statement adheres to our policy

All data, the code used for analysis, as well as a video of the stimulus can be found on Open Science Foundation (https://osf.io/pvmyh/).

Research involving human participants, their data, or biological material

Policy information about studies with human participants or human data. See also policy information about sex, gender (identity/presentation), and sexual orientation and race, ethnicity and racism.

Reporting on sex and gender	We have analyzed the data in terms of sex/gender of our participants (reported in Appendix A) and found that our data did not allow to draw conclusions and sex/gender differences.
Reporting on race, ethnicity, or other socially relevant groupings	-
Population characteristics	We have described the astronaut and control samples in terms of their age and gender.
Recruitment	The participants were recruited through NASA, CSA, ESA and JAXA.
Ethics oversight	The local York University ethics board, the Canadian Space Agency, NASA

Note that full information on the approval of the study protocol must also be provided in the manuscript.

Field-specific reporting

Please select the one below that is the best fit for your research. If you are not sure, read the appropriate sections before making your selection.

☐ Life sciences ☒ Behavioural & social sciences ☐ Ecological, evolutionary & environmental sciences

For a reference copy of the document with all sections, see nature.com/documents/nr-reporting-summary-flat.pdf

Behavioural & social sciences study design

All studies must disclose on these points even when the disclosure is negative.

Study description	quantitative-experimental
Research sample	astronauts and age-matched controls from the local population in Toronto
Sampling strategy	We recruited participants through CSA, NASA, JAXA
Data collection	We used a computer and an Oculus CV1 to present the stimulus. Participants gave their responses using a finger mouse.
Timing	Data collection began on 2018-08-07 and was concluded on 2023-01-09.
Data exclusions	Few data points were excluded from the analysis using the interquartile range. More details can be found in the manuscript.
Non-participation	We do not have data on how many astronauts declined to participate in our study. Two controls we had recruited dropped out due to motion sickness (see manuscript for details).
Randomization	All participants received all experimental conditions.

Reporting for specific materials, systems and methods

We require information from authors about some types of materials, experimental systems and methods used in many studies. Here, indicate whether each material, system or method listed is relevant to your study. If you are not sure if a list item applies to your research, read the appropriate section before selecting a response.

Materials & experimental systems

n/a	Involved in the study
☒	☐ Antibodies
☒	☐ Eukaryotic cell lines
☒	☐ Palaeontology and archaeology
☒	☐ Animals and other organisms
☒	☐ Clinical data
☒	☐ Dual use research of concern
☒	☐ Plants

Methods

n/a	Involved in the study
☒	☐ ChIP-seq
☒	☐ Flow cytometry
☒	☐ MRI-based neuroimaging

Plants

Seed stocks	-
Novel plant genotypes	-
Authentication	-
